# Regulation of the ER Stress Response by the Ion Channel Activity of the Infectious Bronchitis Coronavirus Envelope Protein Modulates Virion Release, Apoptosis, Viral Fitness, and Pathogenesis

**DOI:** 10.3389/fmicb.2019.03022

**Published:** 2020-01-24

**Authors:** Shumin Li, Lixia Yuan, Guo Dai, Rui Ai Chen, Ding Xiang Liu, To Sing Fung

**Affiliations:** ^1^Guangdong Province Key Laboratory of Microbial Signals & Disease Control, and Integrative Microbiology Research Centre, South China Agricultural University, Guangzhou, China; ^2^College of Veterinary Medicine, South China Agricultural University, Guangzhou, China; ^3^Zhaoqing DaHuaNong Biology Medicine Co., Ltd., Zhaoqing, China

**Keywords:** coronavirus, viroporin, ion channel activity, ER stress response, pro-inflammatory cytokine, pathogenesis

## Abstract

Coronavirus (CoV) envelope (E) protein is a small structural protein critical for virion morphogenesis and release. The recently characterized E protein ion channel activity (EIC) has also been implicated in modulating viral pathogenesis. In this study, we used infectious bronchitis coronavirus (IBV) as a model to study EIC. Two recombinant IBVs (rIBVs) harboring EIC-inactivating mutations – rT16A and rA26F – were serially passaged, and several compensatory mutations were identified in the transmembrane domain (TMD). Two rIBVs harboring these putative EIC-reverting mutations – rT16A/A26V and rA26F/F14N – were recovered. Compared with the parental rIBV-p65 control, all four EIC mutants exhibited comparable levels of intracellular RNA synthesis, structural protein production, and virion assembly. Our results showed that the IBV EIC contributed to the induction of ER stress response, as up-regulation of ER stress-related genes was markedly reduced in cells infected with the EIC-defective mutants. EIC-defective mutants also formed smaller plaques, released significantly less infectious virions into the culture supernatant, and had lower levels of viral fitness in cell culture. Significantly, all these defective phenotypes were restored in cells infected with the putative EIC revertants. EIC mutations were also implicated in regulating IBV-induced apoptosis, induction of pro-inflammatory cytokines, and viral pathogenicity *in vivo*. Taken together, this study highlights the importance of CoV EIC in modulating virion release and various aspects of CoV – host interaction.

## Introduction

Coronaviruses (CoVs) are a group of enveloped viruses with non-segmented, single-stranded, and positive-sense RNA genomes (Masters, 2006). Besides infecting a wide range of domesticated and laboratory vertebrates, six human CoVs have been identified, causing respiratory diseases with mild to severe outcomes. Among these, severe acute respiratory syndrome coronavirus (SARS-CoV) and Middle East respiratory syndrome coronavirus (MERS-CoV) are both zoonotic and highly pathogenic CoVs that have emerged in recent epidemic and/or pandemic outbreaks (Li et al., 2005; de Groot et al., 2013).

Coronaviruses have huge RNA genomes ranging from 27,000 to 32,000 nucleotides. The first 2/3 of the genome encodes the viral replicase, while the remaining 1/3 contains coding sequence for the accessory proteins and the four structural proteins: spike (S), envelope (E), membrane (M), and nucleocapsid (N) protein (Masters, 2006). Among them, the S protein is responsible for binding to the cognate receptor and mediating membrane fusion (Yamada and Liu, 2009; Yamada et al., 2009; Zheng et al., 2018); the M protein is crucial for the assembly and morphogenesis of mature virions (De Haan et al., 2000; Liang et al., 2019); the N protein binds to the RNA genome in a beads-on-a-string fashion to form the helically symmetric nucleocapsid (Masters, 2006).

Translated from the subgenomic mRNA3 via an internal ribosomal entry mechanism, the E protein is a small (8–12 kDa), integral membrane protein present at a low amount in the virion (Liu and Inglis, 1991; Liu et al., 1991). Nonetheless, CoV E protein serves an important role in the process of particle assembly, presumably mediated by its ability to induce membrane curvature and its physical interaction with the M protein (Lim and Liu, 2001; Liu et al., 2007). In fact, co-expression of CoV M and E protein is both necessary and sufficient for the production of virus-like particles (VLPs) in cell culture (Bos et al., 1996; Vennema et al., 1996). For some CoVs such as mouse hepatitis virus (MHV) and SARS-CoV, deletion of the E gene did not completely abolish replication, but the virions were severely crippled with significantly reduced titers (Kuo and Masters, 2003; DeDiego et al., 2007). Compared with the wild-type control, SARS-CoV lacking the E gene (rSARS-CoV-ΔE) was also significantly attenuated *in vivo*, presumably due to its deficiency in suppressing host stress response and apoptosis induction (DeDiego et al., 2007, 2011). Recently, the PDZ (Postsynaptic density 95, PSD-85; Disks large, Dlg; Zonula occludens-1, ZO-1)-binding motif (PBM) at the C-terminus of SARS-CoV E protein was demonstrated to interact with a host PDZ protein called syntenin and lead to its relocation to the cytoplasm, thereby activating the p38 kinase to induce the expression of proinflammatory cytokines (Jimenez-Guardeño et al., 2014). Therefore, E protein modulates both viral replication and pathogenesis during CoV infection.

Membrane permeabilizing activity was first identified for the SARS-CoV E protein when it was expressed in *Escherichia coli* and in mammalian cells (Liao et al., 2004, 2006; Yuan et al., 2006). Further structural and biophysical studies have gradually established a cation channel model constituted by the pentameric α-helical bundle of the transmembrane domain (TMD), which exhibited voltage-independent ion conductance regulated by lipid charges (Torres et al., 2005, 2006, 2007; Verdiá-Báguena et al., 2012). For SARS-CoV, two mutations in the TMD – N15A and V25F – have been shown to abolish E protein ion channel activity (EIC) (Verdiá-Báguena et al., 2012). Recombinant SARS-CoV harboring either of these two EIC-defective mutations replicated similarly as the wild-type control in cell culture, but the *in vivo* pathogenicity was dramatically reduced, presumably due to a lower level of calcium efflux and inflammasome activation in the infected cells (Nieto-Torres et al., 2014, 2015). However, several studies have shown that the SARS-CoV accessory protein 3a and 8a also harbor ion channel activity (Lu et al., 2006; Chen et al., 2011), and recombinant SARS-CoV was not viable only when both the E and 3a gene were deleted (Castaño-Rodriguez et al., 2018). This functional redundancy has complicated the mechanistic characterization of EIC in SARS-CoV.

Infectious bronchitis virus (IBV) is a gammacoronavirus infecting chicken. Apart from the E protein, no other IBV protein has been reported to encode ion channel (IC) activity. Two point mutations in the IBV E protein – T16A and A26F – are homologous to the N15A and V25F in the SARS-CoV E protein, respectively (Ruch and Machamer, 2012). The T16A mutation abolished the ability of IBV E protein to disrupt the Golgi complex and host secretory pathway, but did not impact virus assembly as determined by the level of VLP production (Ruch and Machamer, 2012). In contrast, IBV E^A26F^ disrupted cellular secretory pathway, but did not support VLP production (Westerbeck and Machamer, 2015). Using a pH-indicating fluorescent protein pHluorin, it was also found that transfection of IBV E or E^A26F^, but not E^T16A^, correlated with an increase of Golgi luminal pH (Westerbeck and Machamer, 2019). The authors thus concluded that IBV EIC facilitated virion release, presumably by neutralizing the Golgi lumen to protect the S protein from premature proteolytic cleavage (Westerbeck and Machamer, 2019). However, these studies were based on overexpression experiments and the IBV EIC was not investigated by reverse genetics in the setting of actual infections. Recently, we reported the successful recovery of recombinant IBVs (rIBVs) harboring these two mutations (To et al., 2017). Compared with the parental control, rT16A and rA26F exhibited similar levels of intracellular viral replication and assembly, but the levels of virion release were significantly reduced (To et al., 2017).

In this study, we make use of five rIBVs to characterize the function of IBV EIC: the parental rIBV-p65, two EIC defective mutants (rT16A and rA26F), and two putative EIC revertant mutants (rT16A/A26V and rA26F/F14N, respectively). Compared with the parental virus, all four EIC mutants exhibited comparable levels of RNA replication, structural protein translation, and intracellular virion assembly. However, the two EIC-inactive mutants formed smaller plaques, released significantly less infectious virions to the supernatant, and had lower levels of viral fitness in cell culture. Remarkably, all these defective phenotypes were restored in cells infected with the putative EIC revertants. Additionally, EIC also contributed to the activation of ER stress response and the induction of pro-inflammatory cytokines during IBV infection. Moreover, IBV EIC mutations modulated apoptosis induction and correlated with virulence in embryonated chicken eggs. Taken together, using authentic infection systems, our data establish the important roles of EIC in regulating CoV infection, CoV – host interactions, and viral pathogenesis.

## Results

### EIC Contributes to Virus Spreading in Cell Culture

**FIGURE 1:**
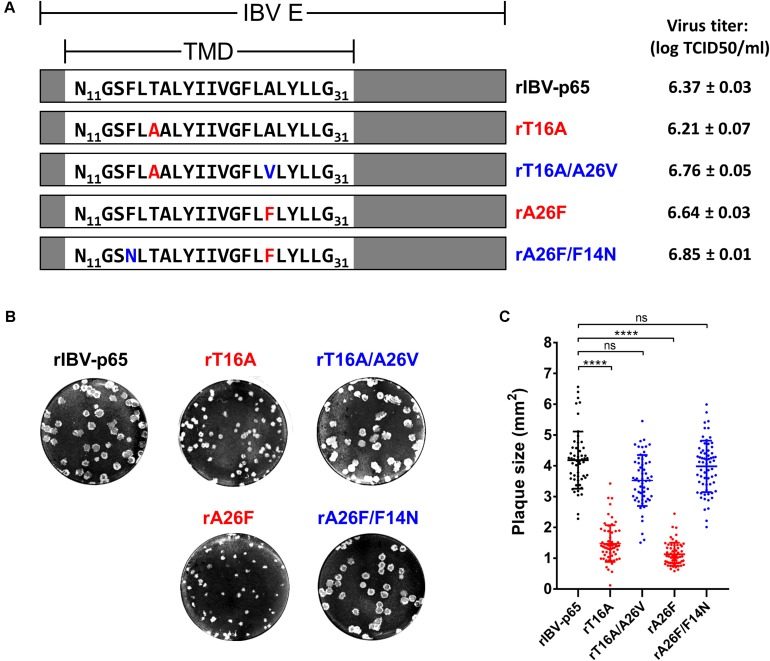
Genotypes and plaque morphologies of rIBV-p65 and the EIC mutants used in this study. **(A)** Genotypes and virus titers of the five rIBVs. Only the amino acid sequences of the transmembrane domain (TMD) of IBV E protein (N11 to G31) were shown. IC-inactivating mutations (T16A and A26F) were in red, while putative IC-reverting mutations (F14N and A26V) were in blue. Titers of the virus stocks were shown in the unit of log TCID50 per ml. **(B)** Representative images showing the plaque morphologies of the above five rIBVs. **(C)** Quantification of plaque sizes of the above five rIBVs. The areas of a minimum of 50 plaques were determined. Comparing with the rIBV-p65 control:     *p* < 0.0001; ns, non-significant.

In this study, the Vero-adapted strain rIBV-p65 was used as the parental control. The two rIBVs harboring channel-inactivating mutations reported previously (To et al., 2017), rT16A and rA26F, were serially passaged in Vero cells. The putative reverting mutation A26V was observed for rT16A in passage 10, and the mutation F14N was observed for rA26F in passage 5 (data not shown). Using reverse genetics, the parental rIBV-p65, the two IC-defective mutants (rT16A and rA26F) and the two corresponding putative revertants (rT16A/A26V and rA26F/F14N) were recovered and plaque-purified. The E gene was sequenced to verify the incorporation of the desired mutations and the absence of additional mutations. The titers of the virus stocks were determined by the TCID50 method. All four mutants achieved high titers comparable to the parental rIBV-p65 with no significant difference among each other (Figure 1A). However, striking differences in the plaque phenotypes were observed. Compared with the parental rIBV-p65, the plaque sizes of the IC-defective mutants were significantly smaller, and the rA26F plaques were apparently even smaller than the rT16A plaques (Figures 1B,C). Unrecognized and not reported in our previous study, such differences in the plaque sizes presumably result from a refined plaque assay protocol with a longer incubation time and the inclusion of 1% FBS in the agarose overlay. Remarkably, plaque sizes of the putative IC revertants returned to similar levels as the parental rIBV-p65 (Figures 1B,C). Taken together, the data suggest that although EIC does not affect the titers of virus stocks, deficiency of EIC results in a distinctive small plaque phenotype.

### EIC Contributes to the Induction of ER Stress Response During IBV Infection

To see whether IBV EIC is involved in the induction of ER stress response, total cellular RNA extracted from infected cells harvested at 24 and 32 h post infection (hpi) were subjected to RT-qPCR analysis for IBV genomic RNA (gRNA) and nine ER stress-related genes. The 24 hpi time point was chosen because the production of intracellular and released viral progeny for rIBV-p65, rT16A, and rA26F peaked at 24 hpi, as determined in our previous study (To et al., 2017). The 32 hpi time point was chosen because upregulation of ER stress-related genes in IBV-infected cells was most significant at the late stage of infection, usually 6–10 h after the peak of viral replication (Liao et al., 2013; Fung et al., 2014b). The host genes included ER stress markers: glucose-regulated protein 78 kDa (GRP78), GRP94, and homocysteine-responsive endoplasmic reticulum-resident ubiquitin-like domain member 1 (HERPUD1); mediator and effectors of the inositol-requiring enzyme 1 (IRE1) branch: total and spliced form of X-box protein 1 (XBP1), ER-associated DNAJ protein 4 (ERdj4), and protein kinase inhibitor p58 (p58^IPK^); protein kinase RNA-like endoplasmic reticulum kinase (PERK) and its downstream transcription factor C/EBP-homologous protein (CHOP).

**FIGURE 2:**
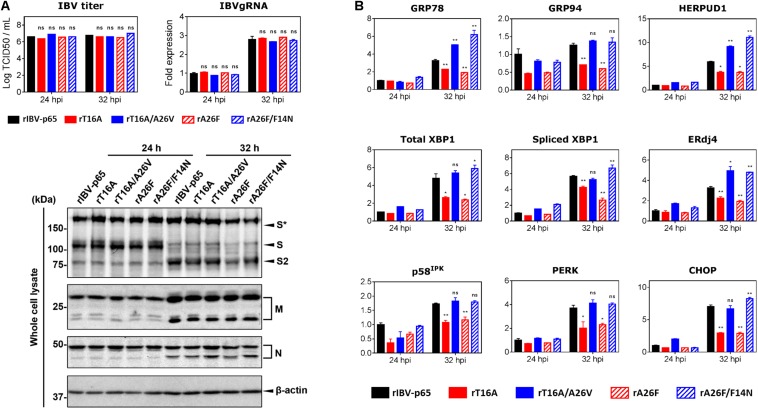
EIC contributes to the upregulation of ER stress-related genes and proinflammatory cytokines during IBV infection. **(A)** Vero cells were infected with the five rIBVs at MOI ∼ 2. Cell lysates were harvested by three freeze/thaw cycles at 24 and 32 h post infection (hpi). Virus titers were expressed in the unit of log TCID50 per ml. Cell lysates were subjected to RNA extraction. Equal amounts of total RNA were reverse transcribed. The levels of IBV genomic RNA (IBVgRNA) were determined by quantitative PCR and normalized to that of the rIBV-p65-infected 24 hpi sample. Cell lysates were also subjected to SDS-PAGE and immunoblotting with antisera against IBV S, M, and N protein. Beta-actin was included as the loading control. Sizes of protein ladders in kDa were indicated on the left. The experiment was repeated three times with similar results, and the result of one representative experiment is shown. S*, glycosylated IBV S protein. Comparing with rIBV-p65-infected sample of the same set: ns, non-significant. **(B)** Cell lysates harvested in **(A)** were subjected to RNA extraction and RT-qPCR analysis to determine the mRNA expression levels of GRP78, GRP94, HERPUD1, total XBP1, spliced XBP1, ERdj4, p58^IPK^, PERK, and CHOP. GAPDH was used as the internal control. Fold changes were determined by normalizing to the rIBV-p65-infected 24 hpi sample. The experiment was repeated three times with similar results, and the result of one representative experiment is shown. Comparing with rIBV-p65-infected sample of the same set: *p < 0.05; **p < 0.01; ns, non-significant.

Vero cells were infected with each of the five abovementioned rIBVs at a multiplicity of infection (MOI) of approximately 2. The amounts of infectious virus particles in the cell lysates were determined by TCID50 assay. As shown in Figure 2A, the virus titers in the lysates of infected cells were similar among the five rIBVs. Comparable amounts of IBV gRNA and IBV structural proteins (S, M, and N) were detected in the cell lysates of Vero cells infected with the five rIBVs. Notably, we have not observed significant difference in the glycosylation and proteolytic processing pattern of the IBV S protein among the five rIBVs. In terms of the M protein, the single and dual glycosylated forms were both clearly visible in cells infected with the five rIBVs.

We then determined the expression levels of host ER stress-related genes at the mRNA level in the IBV-infected cells harvested at 24 and 32 hpi, respectively. Compared with the 24 hpi sample, the mRNA levels of most genes (except GRP94 and p58^IPK^) were upregulated by at least threefold in cells infected with rIBV-p65 at 32 hpi (Figure 2B), suggesting that ER stress response was duly induced at the late stage of IBV infection. At 32 hpi, the mRNA levels of all nine genes were significantly lower in cells infected with rT16A or rA26F, compared with the rIBV-p65 control (Figure 2B). In sharp contrast, the transcription levels of these genes in cells infected with rT16A/A26V or rA26F/F14N were comparable to or even higher than the rIBV-p65 control at 32 hpi (Figure 2B). The data suggest that EIC was involved in the activation of ER stress response during the late stage of IBV infection.

### IC-Reverting Mutations Restore the Defective Virion Release of IBV EIC Mutants

**FIGURE 3:**
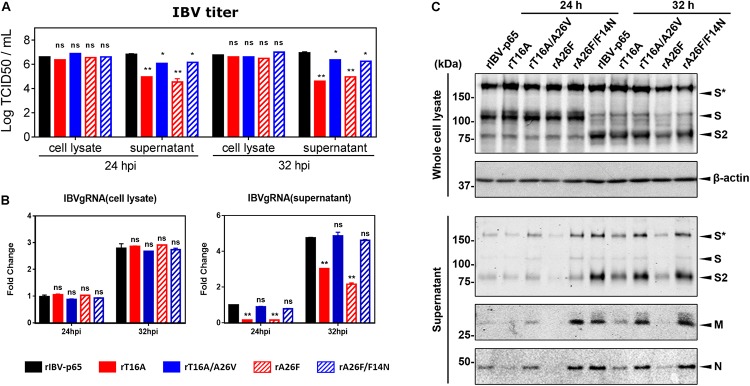
IBV E protein IC activity facilitates the release of infectious virions. **(A)** Vero cells were infected with the five rIBVs at MOI ∼ 2. Cell lysates and supernatant samples were harvested at 24 and 32 hpi. Virus titers were expressed in the unit of log TCID50 per ml. The experiment was repeated three times with similar results, and the result of one representative experiment is shown. Comparing with rIBV-p65-infected sample of the same set: *p < 0.05; **p < 0.01; ns, non-significant. **(B)** Cell lysates and supernatant samples harvested in **(A)** were subjected to RNA extraction. Equal amounts of total RNA were reverse transcribed. The levels of IBV genomic RNA (IBVgRNA) were determined by quantitative PCR and normalized to that of the rIBV-p65-infected 24 hpi sample. The experiment was repeated three times with similar results, and the result of one representative experiment is shown. Comparing with rIBV-p65-infected sample of the same set: **p < 0.01; ns, non-significant. **(C)** Cell lysates and supernatant samples harvested in **(A)** were subjected to SDS-PAGE and Western blot analysis with antisera against IBV S, M, and N protein. Beta-actin was included as the loading control. Sizes of protein ladders in kDa were indicated on the left. The experiment was repeated three times with similar results, and the result of one representative experiment is shown. S*, glycosylated IBV S protein.

Previously, we have shown that virion release is defective in rIBV harboring IC-inactivating mutation in the E gene (To et al., 2017). To determine the effect of the compensatory mutations on virion release, Vero cells were infected with each of the five abovementioned rIBVs at MOI ∼ 2. The amounts of infectious virus particles in the cell lysates and the supernatants were determined by TCID50 assay. As shown in Figure 3A, the intracellular virus titers were similar among the five rIBVs, suggesting that the assembly of infectious virions was not significantly affected by the EIC. In sharp contrast, the supernatant virus titers of rT16A and rA26F were significantly lower by more than 1 log10 compared with that of the parental rIBV-p65. On the other hand, the supernatant virus titers of rT16A/A26V or rA26F/F14N, although lower than that of rIBV-p65, were considerably higher than those of the corresponding IC-inactivating mutants (Figure 3A).

To validate the virus quantification result, the amounts of intracellular and supernatant IBV gRNA were also determined. As shown in Figure 3B, comparable amounts of IBV gRNA were detected inside Vero cells infected with the five rIBVs; thus, viral RNA synthesis was not significantly affected by the EIC. Similar to the virus quantification data, the levels of IBV gRNA were much lower in the supernatant of cells infected with rT16A or rA26F, compared with the rIBV-p65 control. In comparison, supernatant IBV gRNA levels of cells infected with rT16A/A26V or rA26F/F14N were comparable to that of the rIBV-p65 control.

The amount of major structural proteins was also determined. As shown in Figure 3C, similar amounts of IBV S protein were detected in the cell lysates of Vero cells infected with the five rIBVs (Figure 3C). In sharp contrast, the levels of IBV S, M, and N protein were lower in the supernatant sample of rT16A and rA26F, whereas similar levels were detected in the corresponding putative IC-revertants compared with the rIBV-p65 control (Figure 3C). Taken together, the EIC of IBV seems not essential for virion assembly, but it may facilitate the release of infectious particles.

### IC-Reverting Mutations Restore the *in vitro* Fitness of IBV EIC Mutants

**FIGURE 4:**
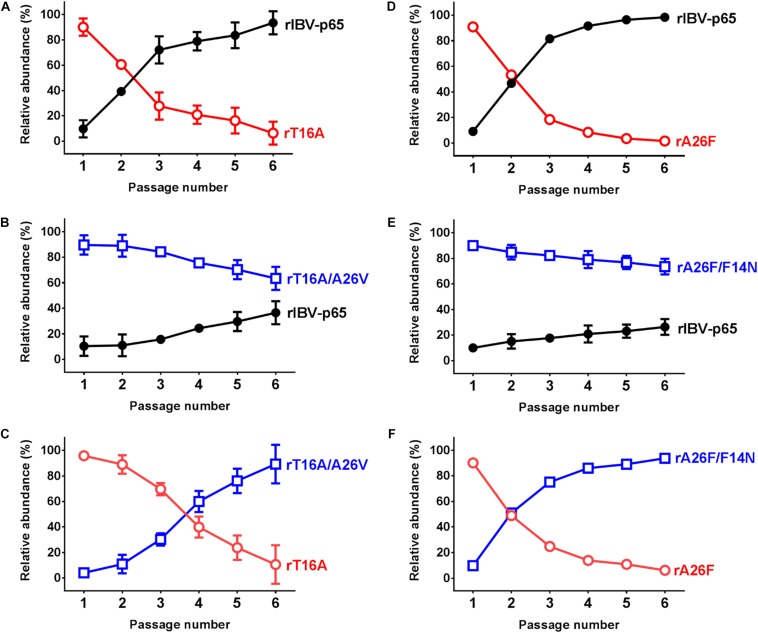
IBV E protein IC activity contributes to viral fitness in cell culture. Competition assays between rIBV-p65 and rT16A **(A)**, rIBV-p65 and rT16A/A26V **(B)**, rT16A/A26V and rT16A **(C)**, rIBV-p65 and rA26F **(D)**, rIBV-p65 and rA26F/F14N **(E)**, rA26F/F14N and rA26F **(F)** were performed in Vero cells coinfected at a ratio of 1:9. The culture supernatant of the infected cells was serially passaged for five times. The relative abundance of each virus was determined by sequencing the IBV E gene as previously described. Error bars show standard deviations from three independent experiments.

Previously we have also shown that the fitness of rIBVs harboring EIC-inactivating mutations was significantly reduced (To et al., 2017). To determine the effect of the EIC-reverting mutations on viral fitness, competition assays were performed between two rIBVs. WT:EIC-defective mutant, WT:revertant, or revertant:EIC-defective mutant was mixed in a ratio of approximately 1:9, and the mixed virus samples were passaged in Vero cells for six times. Consistent with our previous data, the fitness of both rT16A and rA26F were much lower than rIBV-p65, so the two IC mutants were rapidly displaced in a few generations (Figures 4A,D). In contrast, although the relative abundance of both rT16A/A26V and rA26V/F14N gradually reduced during the competition assay, neither of them was outcompeted by rIBV-p65 at passage 6 (Figures 4B,E). Therefore, the fitness of the two EIC-reverting mutants was only slightly lower than that of the parental virus. In line with this, when the EIC-defective mutants were mixed with their corresponding revertants, both IC-defective mutants rapidly lost out in the competition (Figures 4C,F). Taken together, the data suggest that the EIC-reverting mutations restored, though not completely, the fitness of the corresponding IBV EIC-defective mutants.

### EIC Mutations Modulate the Induction of Apoptosis in IBV-Infected Cells

**FIGURE 5:**
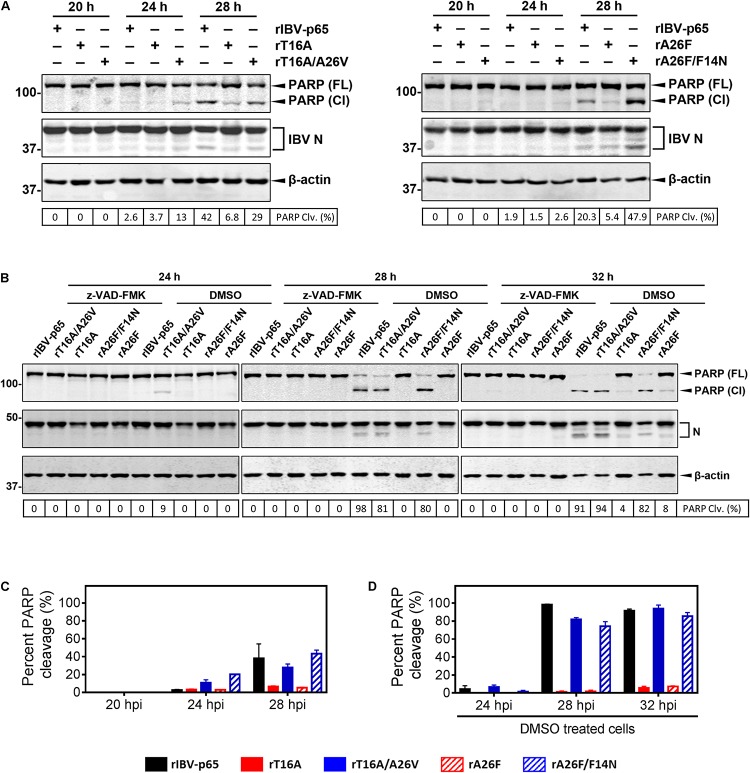
IBV E protein IC mutations modulate apoptosis induction in IBV-infected cells. **(A)** Vero cells were infected with rIBV-p65, rT16A, rT16A/A26V, rA26F, or rA26F/F14N at MOI ∼ 2. Protein samples were harvested at 20, 24, and 28 hpi and subjected to SDS-PAGE and Western blot analysis using the indicated antisera or antibodies. Beta-actin was included as the loading control. Sizes of protein ladders in kDa were indicated on the left. Degree of cell apoptosis was calculated as the band intensity of cleaved PARP protein divided by the band intensity of the total PARP protein. The experiment was repeated three times with similar results **(C)**, and the result of one representative experiment is shown. PARP(FL), full-length PARP; PARP(Cl), cleaved PARP; PARP Clv. (%), percent PARP cleavage. **(B)** Vero cells were infected with rIBV-p65, rT16A/A26V, rT16A, rA26F/F14N, or rA26F at MOI ∼ 2. After 2 h of virus adsorption, cells were incubated with DMEM containing 10 μM of the pan-caspase inhibitor z-VAD-FMK or the same amount of the DMSO solvent control. Protein samples were harvested at 24, 28, and 32 hpi and subjected to SDS-PAGE and Western blot analysis as in **(A)**. Degree of cell apoptosis was calculated as in **(A)**. The experiment was repeated three times with similar results **(D)**, and the result of one representative experiment is shown. PARP(FL), full-length PARP; PARP(Cl), cleaved PARP; PARP Clv. (%), percent PARP cleavage. **(C)** The average and SD values of percent PARP cleavage in **(A)** from three independent experiments. **(D)** The average and SD values of percent PARP cleavage in the DMSO-treated cells in **(B)** from three independent experiments.

Infectious bronchitis coronavirus infection induces caspase-dependent and p53-independent apoptosis in the infected cells (Liu et al., 2001; Li et al., 2007). Moreover, IBV-induced apoptosis is regulated by a number of interrelated host proteins such as c-Jun N-terminal kinase (Fung and Liu, 2017), as well as CHOP and IRE from the UPR pathways (Liao et al., 2013; Fung et al., 2014b). To see whether EIC is implicated in IBV-induced apoptosis, Vero cells were infected with rIBV-p65, rT16A, or rT16A/A26V at MOI ∼ 2 and harvested at 20, 24, and 28 hpi. As shown in Figure 5A, similar amounts of IBV N protein were detected at the same time points for the three rIBVs, suggesting comparable levels of IBV infection. The percentage cleavage of poly (ADP-ribose) polymerase (PARP), a substrate of active caspase 3, was used to determine the level of apoptosis induction. As expected, significant PARP cleavage was detected in cells infected with rIBV-p65 at 28 hpi. Induction of apoptosis was also revealed by the increased cleavage level of N protein, which is also known to be a substrate of effector caspases. In comparison, a very low level and a moderate level of PARP cleavage were observed in cells infected with rT16A and rT16A/A26V at 28 hpi, respectively (Figures 5A,C).

In another set of experiment, apoptosis induction was examined in cells infected with rA26F or rA26F/F14N. Comparable levels of IBV infection were determined by the similar amounts of IBV N protein (Figure 5A). Similarly, infection with rA26F resulted in a significantly lower level of PARP cleavage compared with that of the rIBV-p65 control, whereas much more pronounced PARP cleavage was observed in the rA26F/F14N-infected cells. Consistent patterns of IBV N cleavage were also observed (Figures 5A,C).

We also explore the effect of caspase inhibition on the apoptosis induction by the five rIBVs. After virus adsorption, cells were incubated with DMEM containing 10 μM of the pan-caspase inhibitor z-VAD-FMK or the same amount of the DMSO solvent control. As shown in Figures 5B,D, in the DMSO-treated control, significant PARP cleavage was detected in cells infected with rIBV-p65, rT16A/A26V, or rA26F/F14N at 24, 28, and 32 hpi. On the other hand, in DMSO-treated cells infected with rT16A or rA26F, no PARP cleavage was detectable at 24 and 28 hpi, and only a very low level of PARP cleavage was observed at 32 hpi. No PARP cleavage could be observed in all the samples treated with z-VAD-FMK, suggesting that caspase inhibition completely abolished IBV infection-induced apoptosis. The result suggests that IBV E protein IC activity was involved in the induction of caspase-dependent apoptosis at the late stage of IBV infection.

### EIC Contributes to the Pathogenesis of IBV in Embryonated Eggs

**FIGURE 6:**
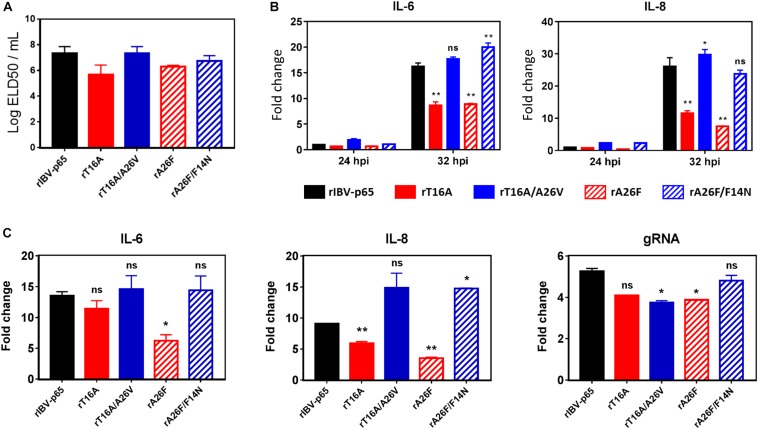
EIC contributes to the pathogenesis of IBV in embryonated eggs and the induction of proinflammatory cytokines. **(A)** Virus stocks of the five rIBVs were 10-fold serially diluted and 0.2 ml diluted virus samples were injected into the allantoic cavities of 10-day old embryonated SPF eggs. The eggs were incubated at 37°C for 5 days. The numbers of live or dead embryos were counted and the 50% embryo lethal dose (ELD50) was calculated using the Reed and Muench method. The bar chart shows the average results from two independent experiments with standard deviations. **(B)** Cell lysates harvested in Figure 2A were subjected to RNA extraction and RT-qPCR analysis to determine the mRNA expression levels of IL-6 and IL-8. GAPDH was used as an internal control. Fold changes were determined by normalizing to the rIBV-p65-infected 24 hpi sample. The experiment was repeated three times with similar results, and the result of one representative experiment is shown. Comparing with rIBV-p65-infected sample of the same set: *p < 0.05; **p < 0.01; ns, non-significant. **(C)** Ten-day-old embryonated chicken eggs were inoculated with 750 PFU per egg of each of the five rIBVs. At 36 hpi, the whole embryos were harvested and homogenized, and total RNA samples were isolated and subjected to RT-qPCR analysis to determine the mRNA expression levels of IL-6, IL-8, and IBV genomic RNA. Fold changes were determined by normalizing to the samples from mock-infected chicken embryos at 36 h post inoculation. The experiment was repeated three times with similar results, and the result of one representative experiment is shown. Comparing with rIBV-p65-infected sample: *p < 0.05; **p < 0.01; ns, non-significant.

Given the effect of IBV EIC on virion release and various aspects of virus – host interactions, we continued to investigate its potential roles in modulating virulence and pathogenesis *in vivo*. To this end, 0.2 ml of diluted virus samples was inoculated into the allantoic cavities of 10-day-old embryonated SPF chicken eggs and the 50% embryo lethal dose (ELD50) was determined. Compared with the rIBV-p65 control, the ELD50 of rT16A and rA26F were both moderately reduced, suggesting slightly lower levels of *in vivo* pathogenicity (Figure 6A). On the other hand, while the EID50 of rT16A/A26V returned to a level comparable to that of the rIBV-p65 control, the EID50 of rA26F/F14N was only marginally higher than that of rA26F. The data suggest that although not essential for viral replication, EIC indeed contributed to the pathogenesis of IBV *in vivo*.

To see whether EIC of IBV is involved in the induction of proinflammatory cytokines, total cellular RNA samples harvested from IBV-infected Vero cells at 24 and 32 hpi were subjected to RT-qPCR analysis for two pro-inflammatory cytokines interleukin-6 (IL-6) and IL-8. Compared with the 24 hpi control, the mRNA levels of IL-6 and IL-8 were upregulated by 15- and 25-fold in cells infected with rIBV-p65 at 32 hpi (Figure 6B), suggesting that transcription of pro-inflammatory cytokines was duly induced at the late stage of IBV infection. At 32 hpi, the mRNA levels of both genes were significantly lower in cells infected with rT16A or rA26F, compared with the rIBV-p65 control. In sharp contrast, the transcription levels of these genes in cells infected with rT16A/A26V or rA26F/F14N were comparable to or even higher than the rIBV-p65 control at 32 hpi (Figure 6B).

To determine the *in vivo* induction of pro-inflammatory cytokines, 10-day-old embryonated chicken eggs were inoculated with 750 PFU per egg of each of the five rIBVs. At 36 hpi, the whole embryos were harvested and homogenized, and total RNA samples were isolated and subjected to RT-qPCR analysis. Mock-infected chicken embryos were used as the normalizing control. As shown in Figure 6C, the mRNA levels of IL-6 and IL-8 were upregulated by approximately 13-fold and 9-fold, respectively, in chicken embryos infected with rIBV-p65, compared with the mock-infected embryos. Induction of IL-8 was slightly reduced in rT16A-infected embryos, whereas induction of both IL-6 and IL-8 was significantly reduced in rA26F-infected embryos, compared with the rIBV-p65 control. In contrast, the mRNA levels of IL-6 in rT16A/A26V- and rA26F/F14N-infected embryos were comparable to the rIBV-p65 control, while the mRNA levels of IL-8 were even higher. When the amounts of IBV gRNA were determined, it was found that the synthesis of IBV genome was slightly reduced in embryos infected with the EIC-defective or EIC reverting mutants, as compared with the rIBV-p65 control. Taken together, the data suggest that IBV E protein IC activity contributes to the induction of pro-inflammatory cytokines at late stage of IBV infection both in cell culture and in chicken embryos.

## Discussion

CoV E protein is a small structural protein that serves critical functions in the morphogenesis and release of mature virions (DeDiego et al., 2007; To et al., 2017), and its recently characterized IC activity has been implicated in modulating viral pathogenesis (Nieto-Torres et al., 2014, 2015). In this study, we have constructed five rIBVs: the parental rIBV-p65, two rIBVs harboring IC-inactivating mutations in the E protein (rT16A and rA26F), and two putative revertants harboring one additional mutation in the TMD of E protein (rT16A/A26V and rA26F/F14N, respectively). Using these five rIBVs, we have shown that, although not essential for intracellular genome replication and virion assembly, the EIC of IBV is required for the efficient release of infectious virions and viral fitness *in vitro*. It is also required for the activation of ER stress and induction pro-inflammatory cytokines during the late stage of IBV infection. Moreover, IC mutations also modulate IBV-induced apoptosis and *in vivo* pathogenesis. Taken together, our data establish the important roles of EIC in regulating CoV infection, CoV – host interactions, and viral pathogenesis.

Previous studies on the CoV EIC have mainly focused on that of the SARS-CoV E protein (Liao et al., 2006; Yuan et al., 2006). These studies have established the structural basis behind the CoV EIC (Torres et al., 2005) and revealed its significance in modulating the pathogenesis of SARS-CoV (Nieto-Torres et al., 2014, 2015). However, several studies have shown that the SARS-CoV accessory protein 3a and 8a also harbor IC activity (Lu et al., 2006; Chen et al., 2011). Moreover, while rSARS-CoV lacking both the E and 3a proteins is not viable (Castaño-Rodriguez et al., 2018), rSARS-CoV lacking the E protein alone is viable, although significantly attenuated *in vitro* and *in vivo* (DeDiego et al., 2007). This functional redundancy complicates the interpretations of phenotypes associated with IC mutations in the E protein, as compensatory functions of the accessory proteins need to be taken into consideration. On the contrary, deletion of E gene is lethal for IBV (unpublished data), and no other proteins encoded by the IBV genome is reported to harbor IC activity. Notably, SARS-CoV E protein IC inactive mutants grow similarly as the parental virus, and their slightly lower fitness is only revealed in a long-term (20 passages) competition assay (Nieto-Torres et al., 2014). In comparison, a significant smaller plaque size, a reduction of released virions by ∼ 1 log10, and a much lower *in vitro* fitness were observed in the IBV E protein IC inactive mutants, while all these defective phenotypes were almost completely restored in the IC-reverting mutants. Taken together, due to the absence of compensatory viroporins, EIC appears to be more critical for the replication of IBV. This also makes IBV a more desirable model to study the specific function of IC activity contributed by the E protein.

Compared with the SARS-CoV E protein, the location and nature of IC-reverting mutations are also remarkably different in the IBV E protein. In SARS-CoV E protein, N15 is predicted to be located facing the channel lumen; its mutation to alanine presumably compromise the attraction of cation but is not likely to affect the channel architecture (Nieto-Torres et al., 2014). In comparison, V25 is located at monomer–monomer interface of the SARS-CoV E protein, and its mutation to a bulkier phenylalanine is likely to introduce larger structural changes to the ion channel (Nieto-Torres et al., 2014). Such speculations agreed well with the IC-reverting mutations observed during serial passages *in vivo* and in culture cells. An *in situ* mutation from alanine to the polar aspartic acid (D) was identified for rSARS-CoV-E-N15A. For rSARS-CoV-E-V25F, multiple compensatory mutations of the V25F-facing TMD residues presumably restored oligomerization and thus channel activity (Nieto-Torres et al., 2014). In our previous study, we have shown that unlike the homologous N15A mutation in SARS-CoV, the T16A mutation in IBV E protein indeed abolished oligomerization, which was partially restored in the T16A/A26V revertant (To et al., 2017). Additionally, IBV E A26F did not abolish oligomerization and was compensated for by mutations at the N- and C-terminal extramembrane domains (To et al., 2017). In this study, we identified yet another reverting mutation for A26F – replacement of the bulky aromatic phenylalanine at position 14 with the polar non-charged asparagine. Although conductance measurement has not been performed for IBV E^*A26F*/*F14N*^, phenotypes of the corresponding rIBV strongly suggested that IC activity should be restored. The reciprocal occurrences of IC-reverting mutations at A26 or at location proximal to T16 suggest certain level of functional and/or structural relatedness. While the pentameric model depicts the general structure of CoV E protein ion channels, conformations and monomer–monomer interactions may vary among different CoV species. Further structural and biophysical analysis are thus required to unravel the mechanisms behind the IC-inactivating and IC-reverting mutations of the IBV E protein.

In terms of virion assembly and release, previous transfection-based studies have shown that although the T16A mutation in IBV E protein abolished its activity to disrupt VSV G glycoprotein trafficking and the morphology of the Golgi complex, production of VLP was not significantly affected (Ruch and Machamer, 2012). In contrast, the A26F mutation in IBV E protein retained the ability to disrupt cellular secretory pathway but did not support VLP production (Westerbeck and Machamer, 2015). In a recent study by the same group, IBV infection, transfection of wild-type IBV E, or transfection of E^A26F^, but not E^T16A^, correlated with an increase of Golgi luminal pH (Westerbeck and Machamer, 2019). The neutralization of Golgi lumen presumably protected IBV S protein from premature cleavage and facilitated the release of infectious virus from the cells (Westerbeck and Machamer, 2019). Evidence from these studies suggests that the two IC-inactivating mutations might affect virion release via different mechanisms. In this study, we have observed reduced levels of both IBVgRNA and structural proteins in the supernatant of cells infected with rT16A or rA26F, compared with the rIBV-p65 control. Such deficiency in virion release seemed to be more pronounced in the rA26F-infected cells, indicating that the A26F mutation might be comparatively more destructive to the EIC of IBV. However, no apparent difference was observed in the intracellular cleavage patterns of IBV S protein in cells infected with the five rIBV, suggesting that IBV S processing was not significantly affected by the EIC. To reveal the mechanistic involvement of EIC in virion release, it would be very meaningful to determine the levels of Golgi neutralization and S incorporation in cells infected with the EIC-defective mutants and respective revertants.

Ca^2+^ is one of the most important secondary messengers, relaying signals for a diversity of cellular activities such as protein synthesis, muscle contraction, cell cycle progression, and apoptosis (Clapham, 2007). Intracellular Ca^2+^ is mainly stored in the ER lumen, where it binds with Ca^2+^-binding protein chaperones (such as calreticulin) to facilitate proper folding of nascent proteins in the ER (Michalak et al., 2009). Therefore, the disturbance of luminal Ca^2+^ concentration can substantially impact the ER folding capacity and activate ER stress response. Previous studies have shown that SARS-CoV E protein can form Ca^2+^ channels in the ERGIC/Golgi membranes (Nieto-Torres et al., 2015) and that disturbance of cellular Ca^2+^ homeostasis may lead to ER stress and cell death (Sano and Reed, 2013). In previous studies, we have shown that IBV infection induces ER stress and activates the PERK/PKR-eIF2α and the IRE1-XBP1 branch of the UPR (Liao et al., 2013; Fung et al., 2014b). In this study, we observed that the upregulation of ER stress-related genes was significantly reduced in the absence of IBV EIC. Although *in vitro* conductance measurement of IBV E was performed with potassium ions (To et al., 2017), it is quite possible that IBV E protein may also form Ca^2+^ channels in the ER, thereby activating ER stress response in the infected cells. Further studies using time lapse Ca^2+^ imaging would be helpful to explore this hypothesis. Alternatively, IBV EIC may also induce ER stress indirectly, presumably by disrupting normal cellular secretory pathway or causing structural alterations to the ER/Golgi membrane network.

By serving as a Ca^2+^ channel, the SARS-CoV E protein boosted the activation of the NLR family pyrin domain containing 3 (NLRP3) inflammasome, thereby contributing to the production of proinflammatory cytokines such as tumor necrosis factor (TNF) and IL-6 (Nieto-Torres et al., 2014, 2015). In this study, the induction of IL-6 and IL-8 was significantly reduced in cells infected with the EIC mutants, but was restored to levels comparable to the rIBV-p65 control in cells infected with the respective revertants. While it would be interesting to examine the involvement of the inflammasome pathway, induction of proinflammatory cytokines may also be mediated by the ER stress pathway. In fact, overexpression of the MHV S protein was shown to induce ER stress and upregulate the transcription level of CXCL2 (mouse homolog of human IL-8) (Versteeg et al., 2007). Other UPR effector proteins are also known to participate in the induction of proinflammatory and innate immune response (Fung and Liu, 2014; Fung et al., 2014a). Similarly, the CoV EIC may either directly or indirectly modulate apoptosis induction. CoV EIC may directly lead to Ca^2+^ efflux, which activates apoptosis by directly depolarizing the inner mitochondrial membrane (Giorgi et al., 2008). Alternatively, CoV EIC may first activate the ER stress and UPR, which then mediates apoptosis via the activation of JNK, CHOP, or caspase 12 (Szegezdi et al., 2006).

In terms of *in vivo* pathogenesis, previous studies have shown that mice infected with recombinant SARS-CoV lacking EIC exhibited less severe lung damage and most survived the infection, compared with the wild-type control that rapidly lost weight and died (Nieto-Torres et al., 2014). Also, revertant SARS-CoVs that restored EIC were shown to regain a virulent phenotype in mice, validating the correlation between SARS-CoV EIC and pathogenicity (Nieto-Torres et al., 2014). In this study, we also observed a link between IBV EIC and virulence in embryonated chicken eggs. Further studies on the *in vivo* viral replication and host immune response are required to elucidate the modulatory mechanisms of IBV EIC.

## Materials and Methods

### Cells and Virus

The egg-adapted Beaudette strain of IBV (ATCC VR-22) was obtained from the American Type Culture Collection (ATCC) and adapted to Vero cells as previously described (Ng and Liu, 1998; Shen et al., 2003, 2004; Fang et al., 2005). Vero cells were cultured in Dulbecco’s modified Eagle’s medium (DMEM, Life Technologies, Carlsbad, CA, United States) supplemented with 6% fetal bovine serum (FBS), 100 U/ml penicillin, and 100 μg/ml streptomycin. Confluent Vero cells were inoculated with IBV at an MOI of approximately 0.1 and cultured in plain DMEM at 37°C, until almost the entire monolayer exhibited cytopathic effect (CPE) in the form of multinucleated syncytia. The virus stocks were harvested by three rounds of freeze–thaw, aliquot in 1.5-ml screw-cap vials and stored at −80°C. The titers of the virus stocks were determined by plaque assays and 50% tissue culture infective dose (TCID50) assay.

### Construction of rIBVs

rIBVs harboring IC inactivating mutations (rT16A and rA26F) were constructed as previously described (To et al., 2017). To generate the IC revertant mutants, additional point mutations [T16A (ACA to GCA)/A26V (GCA to GTA) and A26F (GCA to TTT)/F14N (TTT to AAC)] were introduced to the E gene in the plasmid pGEM-IBV (20897-27614). The rIBVs were recovered as previously described (Fang et al., 2007). Briefly, five genome fragments were excised from individual plasmid vectors using type IIS restriction endonuclease *Bsm*BI or *Bsa*I. The fragments were then joined by seamless assembly into the full-length IBV cDNA genome containing a 5′-T7 promoter and a 3′-polyadenylate tail. Using this as the template, full length IBV genomic RNA was synthesized by *in vitro* transcription using the mMessage mMachine T7 kit (Ambion, Austin, TX, United States) according to the manufacturer’s instructions. The IBV N transcript was similarly synthesized with a linearized plasmid template. The full length IBV genome and N transcripts were then electroporated into Vero cells by 100 V square waves for 25 ms using the Gene Pulser X-cell electroporation system (Bio-Rad). The cells were incubated in DMEM supplemented with 1% FBS overnight. The medium was then changed to plain DMEM, and the cells were monitored for the appearances of CPE in the next 48–96 h. The rIBVs were purified by at least two rounds of plaque purification.

### Plaque Assay and Determination of Plaque Size

For plaque assay, virus samples were 10-fold serially diluted using serum-free medium, typically 10–10^5^ for supernatant samples and 10^2^–10^6^ for cell lysates. Confluent monolayers of Vero cells seeded on 6-well plates were washed once with plain DMEM, and 200 μl of diluted virus sample was added to each well. The plates were agitated every 10–15 min to ensure proper coverage. After 2 h of adsorption, unbound viruses were removed and cells were washed once with plain DMEM. Two milliliters of overlay medium (0.4% agarose in DMEM containing 1% FBS) was added to each well and the plates were incubated at 37°C for 3 days before plaques formed. Agarose overlay was removed and cells were fixed with 4% formaldehyde before staining with crystal violet. Finally, plaque numbers were counted and virus titer was expressed as logarithm of plaque-forming units (PFU) per ml. Each sample was titrated in triplicate in each experiment. To determine plaque size, a plaque was selected by the freehand selection tool and the area was measured using the NIH ImageJ software. The sizes of at least 50 plaques were measured for each sample.

### Virus Titration by TCID50

Supernatant samples were harvested from IBV-infected cells and clarified by centrifugation at 16,000 × *g* at 4°C for 5 min. Cell lysates were harvested by subjecting IBV-infected cells to three freeze–thaw cycles and clarified by centrifugation at 16,000 × *g* at 4°C for 5 min. Virus samples were kept at −80°C for less than 2 weeks before the titration experiment. Virus titer was determined by the tissue culture infective dose 50 (TCID50) assay. Briefly, virus samples were 10-fold serially diluted. Confluent monolayers of Vero cells seeded on 96-well plates were washed once with plain DMEM, and 100 μl of diluted virus sample was added to each well, with 8 wells used for each dilution. Cells were incubated at 37°C for 3–5 days and examined with a phase contrast microscope. Wells were determined as either positive (with CPE) or negative (without CPE), and TCID50 was calculated using the Reed and Muench method (Reed and Muench, 1938). The virus titer was expressed in the unit of logarithm of TCID50 per ml. Each sample was titrated in duplicate or triplicate in each experiment.

### SDS-PAGE and Western Blot Analysis

To obtain whole cell lysates for protein analysis, cells were harvested at the indicated time points using cell scrapers (Corning) and collected by centrifugation at 16,000 × *g* for 1 min. The supernatant was discarded, and the cell pellet was lysed in 1 × RIPA buffer (10 mM Tris–HCl, pH 8.0, 140 mM NaCl, 0.1% SDS, 1% Triton X-100, 0.1% sodium deoxycholate, 1 mM EDTA, and 0.5 mM EGTA). After clarified by centrifugation, the protein concentration of the cell lysate was determined. The cell lysate was then mixed with 5 × Laemmli sample buffer (0.3125 M Tris–HCl, pH 6.8, 10% SDS, 50% glycerol, 25% β-mercaptoethanol, and 0.025% bromophenol blue) (Laemmli, 1970). On the other hand, culture supernatant was clarified by brief centrifugation and mixed with 5 × Laemmli sample buffer. Protein samples were boiled at 90°C for 5 min and centrifuged at 16,000 × *g* for 5 min. Equal amounts of protein samples were loaded to each well and separated by sodium dodecyl sulfate–polyacrylamide gel electrophoresis (SDS-PAGE) using the Bio-Rad Mini-PROTEAN Tetra cell system. The resolved proteins were then transferred to a 0.2-μm nitrocellulose membrane using the Bio-Rad *Trans*-Blot protein transfer system. To block off non-specific binding sites, the membrane was incubated with 5% skim milk in 1 × TBST buffer (20 mM Tris–HCl, pH 7.4, 150 mM NaCl, and 0.1% Tween 20) at room temperature for 1 h. The membrane was then incubated with 1 μg/ml specific primary antibody dissolved in 1 × TBST with 3% BSA (w/v) at 4°C overnight. The membrane was washed three times with 1 × TBST, and incubated with 1:10,000 diluted IRDye 800CW goat anti-rabbit or 680RD goat anti-mouse IgG secondary antibodies (Licor) at room temperature for 2 h. The membrane was washed three times with 1 × TBST, and fluorescence imaging was performed using the Azure c600 Imager according to the manufacturer’s instruction. Densitometric measurement was performed using the AzureSpot software. All experiments were repeated for at least three times with similar result, and one of the representative results was shown.

### Genetic Stability Through Serial Passaging

Confluent monolayers of Vero cells seeded on the 35-mm^2^ dishes were infected with rIBV-p65, rT16A, rA26F, rT16A/A26V, or rA26F/F14N at MOI ∼ 2. When the infected cells approached complete CPE, culture supernatants were collected and passaged on fresh monolayers of Vero cells. The rIBVs were serially passaged for 30 times, and the E gene was sequenced at passages 0, 5, 10, 15, 20, 25, and 30. Briefly, total RNA samples were extracted from the infected cells and reverse transcribed. The IBV genome region spanning nucleotides 23944 to 24693 was amplified using the forward primer 5′-CGCTCCAACAACTAATACAAG-3′ and the reverse primer 5′-AATGTTAAGGGGCCAAAAGC-3′. The PCR product was sequenced using the primer 5′-TTGAAACTGGTGAGCAAGTGA-3′.

### Virus Competition Assays

Vero cells were co-infected with rIBV-p65 and mutant rIBVs at a ratio of 1:9. Alternatively, cells were co-infected with the revertant mutant and its corresponding IC-inactivating mutant at a ratio of 1:9. When the infected cells approached complete CPE, culture supernatants were collected and passaged on fresh monolayers of Vero cells. The viruses were serially passaged for six times, and the E gene was sequenced at each passage as described above (To et al., 2017). The relative abundance of rIBV-p65, the IC-inactivating mutants, and the revertant mutants was determined as previously described (To et al., 2017).

### RNA Extraction and RT-qPCR Analysis

Total RNA was extracted using the TRIzol reagent (Invitrogen) according to the manufacturer’s instructions. Briefly, cells were lysed with 1 ml of TRIzol per 10 cm^2^ effective growth area, and the lysates were vigorously mixed with one-fifth volume of chloroform. The mixture was then centrifuged at 12,000 × *g* at 4°C for 15 min, and the aqueous phase was mixed with an equal volume of isopropanol. The RNA was precipitated by centrifugation at 12,000 × *g* at 4°C for 15 min, washed twice with 70% ethanol, and dissolved in 30–50 μl of RNase-free water. The total RNA was reverse transcribed using the FastKing gDNA Dispelling RT SuperMix kit (Tiangen) according to the manufacturer’s instructions. Briefly, 2 μg of total RNA was mixed with 4 μl 5 × FastKing-RT SuperMix (containing RT enzyme, RNase inhibitor, random primers, oligo dT primer, dNTP, and reaction buffer) in a 20-μl reaction mixture. Using a thermo cycler, reverse transcription was performed at 42°C for 15 min and the RT enzyme was then inactivated at 95°C for 3 min. The cDNA was then diluted 20-fold with RNase-free water for quantitative PCR (qPCR) analysis, using the Talent qPCR PreMix SYBR Green kit (Tiangen) according to the manufacturer’s instructions. Briefly, 8.4 μl of diluted cDNA was mixed with 10 μl of 2 × qPCR PreMix, 0.4 μl of 50 × ROX, 0.6 μl of 10 μM forward primer, and 0.6 μl of 10 μM reverse primer for a 20-μl reaction mixture. The qPCR analysis was performed using a QuantStudio 3 Real-Time PCR System (Applied Biosystems). The standard protocol included enzyme activation at 50°C for 3 min, initial denaturation at 95°C for 3 min, followed by 40 cycles of denaturing (95°C, 5 s) and annealing/extension (60°C, 30 s) with fluorescent acquisition at the end of each cycle. The results obtained were in the form of cycle threshold (C_T_) values. Using the ΔΔC_T_ method, the relative abundance of a transcript was calculated using GAPDH as an internal control and normalized to the rIBV-p65 control. The following qPCR primers (forward and reverse) were used: GAPDH, 5′-CTGGGCTACACTGAGCACC-3′ and 5′-AAGTGGTCGTTGAGGGCAATG-3′; IBVgRNA, 5′-GTTCTCGCATAAGGTCGGCTA-3′ and 5′-GCTCACTAAA CACCACCAGAAC-3′; IBVsgRNA2, 5′-GCCTTGCGCTAGA TTTTTAACTG-3′ and 5′-AGTGCACACAAAAGAGTCACTA-3′; GRP78, 5′-GAAAGAAGGTTACCCATGCAGT-3′ and 5′-CA GGCCATAAGCAATAGCAGC-3′; GRP94, 5′-GCTGACGATGA AGTTGATGTGG-3′ and 5′-CATCCGTCCTTGAGCCTTCT CTA-3′; HERPUD1, 5′-ATGGAGTCCGAGACCGAAC-3′ and 5′-TTGGTGATCCAACAACAGCTT-3′; total XBP1, 5′-CCCT CCAGAACATCTCCCCAT-3′ and 5′-ACATGACTGGGTCCA AGTTGT-3′; spliced XBP1, 5′-TGCTGAGTCCGCAGCAGG TG-3′ and 5′-ACATGACTGGGTCCAAGTTGT-3′; ERdj4, 5′- TCTTAGGTGTGCCAAAATCGG-3′ and 5′-TGTCAGGGTGG TACTTCATGG-3′; p58^*IPK*^, 5′-GGATGCAGAACTACGGGAA CT-3′ and 5′-TCTTCAACTTTGATGCAGCTT-3′; PERK, 5′-GG AAACGAGAGCCGGATTTATT-3′ and 5′-ACTATGTCCATTA TGGCAGCTTC-3′; CHOP, 5′-GAACGGCTCAAGCAGGAAA TC-3′ and 5′-TTCACCATTCGGTCGATCAGAG-3′; IL-6, 5′-GT GCAAATGAGTACAAAAGTCCTGA-3′ and 5′-GTTCTGCGC CTGCAGCTTC-3′; IL-8, 5′-AAGACGTACTCCAAACCTAT CCAC-3′ and 5′-TCTGTATTGACGCAGTGTGGTC-3′.

### Determination of the ELD50

The virus stocks were 10-fold serially diluted with DMEM, and 200 μl of diluted virus solution was injected into the allantoic cavity of 10-day-old embryonated specific pathogen-free (SPF) chicken eggs. Five eggs were used for each dilution. The eggs were then incubated at 37°C for 5 days, transferred to a 4°C refrigerator, and incubated overnight. The chicken embryos were extracted and examined, and the number of dead embryos was counted. The ELD50 was calculated using the Reed and Muench method (Reed and Muench, 1938).

### Statistical Analysis

The one-way ANOVA method was used to analyze the significant difference between the indicated sample and the respective control sample. Significance levels were presented by the *p*-value (ns, non-significant; ^∗^*p* < 0.05; ^∗∗^*p* < 0.01; ^*⁣*⁣**^p < 0.0001).

## Data Availability Statement

The datasets generated for this study are available on request to the corresponding author.

## Author Contributions

DL and TF designed and organized the study. SL did most of the experimental work. LY and GD did part of the experimental work. RC analyzed the data. SL, TF, and DL wrote the manuscript.

## Conflict of Interest

RC was employed by the company Zhaoqing DaHuaNong Biology Medicine Co., Ltd. The remaining authors declare that the research was conducted in the absence of any commercial or financial relationships that could be construed as a potential conflict of interest.
